# Congruity of genomic and epidemiological data in modelling of local cholera outbreaks

**DOI:** 10.1098/rspb.2023.2805

**Published:** 2024-03-20

**Authors:** Mateusz Wilinski, Lauren Castro, Jeffrey Keithley, Carrie Manore, Josefina Campos, Ethan Romero-Severson, Daryl Domman, Andrey Y. Lokhov

**Affiliations:** ^1^ Theoretical Division, Los Alamos National Laboratory, Los Alamos, NM, USA; ^2^ Analytics, Intelligence and Technology Division, Los Alamos National Laboratory, Los Alamos, NM, USA; ^3^ Department of Computer Science, University of Iowa, Iowa City, IA, USA; ^4^ UO Centro Nacional de Genómica y Bioinformtica, ANLIS ‘Dr. Carlos G. Malbrán, Buenos Aires, Argentina; ^5^ Center for Global Health, Department of Internal Medicine, University of New Mexico Health Sciences Center, Albuquerque, NM, USA

**Keywords:** epidemic modelling, cholera, model selection, genomic data‌

## Abstract

Cholera continues to be a global health threat. Understanding how cholera spreads between locations is fundamental to the rational, evidence-based design of intervention and control efforts. Traditionally, cholera transmission models have used cholera case-count data. More recently, whole-genome sequence data have qualitatively described cholera transmission. Integrating these data streams may provide much more accurate models of cholera spread; however, no systematic analyses have been performed so far to compare traditional case-count models to the phylodynamic models from genomic data for cholera transmission. Here, we use high-fidelity case-count and whole-genome sequencing data from the 1991 to 1998 cholera epidemic in Argentina to directly compare the epidemiological model parameters estimated from these two data sources. We find that phylodynamic methods applied to cholera genomics data provide comparable estimates that are in line with established methods. Our methodology represents a critical step in building a framework for integrating case-count and genomic data sources for cholera epidemiology and other bacterial pathogens.

## 1. Introduction

Cholera is a major public health threat with an estimated 4 million cases a year and over 150 000 deaths annually [1]. Cholera is an acute diarrhoeal disease caused by toxigenic *Vibrio cholerae*, and is transmitted though the faecal–oral route from contaminated food or water. Currently, the burden of disease is primarily in sub-Saharan Africa and South Asia, in vulnerable populations with a lack of access to clean drinking water and sanitation [2]. Of note, some of the largest cholera epidemics have occurred since the 1990s. Case counts over 1 million were documented for the 1991–1998 epidemic in Latin America [3], over 800 000 cases in Haiti from 2010 to 2020 [4], and over 2.5 million cases thus far in the ongoing epidemic in Yemen that began in 2016 [5].

Understanding how cholera is transmitted within and across populations is paramount to the rational design and implementation of control efforts. One of the difficulties in traditional modelling of cholera outbreaks with case-count data alone is that the inference results strongly depend on the quality of the surveillance and reporting procedures, while detailed properties of statistical counting noise are often unknown and cannot be easily estimated. Furthermore, accurate case counting is hampered by the large heterogeneity in disease presentation which ranges from asymptomatic to severe cholera [2]. Genetic sequence data offer another data source on transmission dynamics that could help develop a new generation of complex, but well-constrained cholera models. This is possible due to the fact that epidemiological processes such as transmission and migration of infection leave a trace in pathogen genetic sequence data by changing the underlying infection genealogy of a sampled set of sequences [6–8]. For example, compared with a stable population, in a population experiencing rapid growth, two randomly selected people will, on average, share a common ancestor in the distant past [9], and therefore be separated by a larger number of mutations. Likewise, in otherwise isolated populations, migration links pathogens through a network of common descent [10] leading to a distinctive pattern of interdigitated sequences in a phylogeny. Therefore, pathogen genetic sequence data generally have the potential to inform epidemiological parameters such as transmission, migration and mixing rates. Previous work has used *Vibrio cholerae* sequence data to describe the broad, qualitative flow of cholera at the global scale that can capture broad trends but not explicit details of the transmission process [11–15]. However, the main challenge in the effort to integrate genomic data into cholera transmission models is the lack of evidence that genomic data are informative of cholera transmission processes at the local scale, i.e. that there is sufficient genetic diversity in a single-source, local cholera outbreak to estimate transmission parameters.

Case counts and the genetic sequence data can be thought of as being two independent observers of the transmission dynamics of cholera. In this paper, we use a rich dataset of both case-count data as well as a large collection of genomic sequencing data from Argentina [16] to model the spread of cholera and specifically address the role of local migration as a driving factor in cholera transmission. Our goal is to build a meta-population model with a minimal number of assumptions which accounts for migration, and understand if the two observers agree in their predictions. The bridging and common constraints on both sources of data will be achieved using yet another source of independent data such as high-fidelity estimation of migration flows.

Our modelling choices are aimed at simplicity and driven by the overall goal of checking the consistency between these data sources. For instance, we deliberately take an agnostic stance on the open questions related to the role of the environment or details of bacterial dynamics: while several previous studies explicitly included the environmental compartment [17–21] leading to a larger number of model parameters, we chose to model cholera dynamics as an *effective* transmission process which includes a periodic functional dependence on seasonality, similarly to the approach of Peak *et al*. [22]. To account for discreteness in observed cases, we propose a novel sampling model which relates the continuous model with the discrete observed case counts.

Most key model parameters will be directly inferred from case counts and migration data. Further, a subset of most important parameters such as transmission amplitudes and fraction of asymptomatic infections are independently inferred from the genomic sequence data. In both cases, we use the same meta-population model, so that we can compare corresponding parameters within their uncertainties. In particular, we do not make any *a priori* quantitative assumptions on the fraction of asymptomatic infections, in previous studies ranging from 1% to more than 90% of the population [23–25]. Instead, we keep this important model parameter free, infer its values from data under different settings and discuss the sensitivity of this parameter to various modelling assumptions. We also provide a series of careful sensitivity studies that study the stability of the inferred parameters related to all of our modelling assumptions.

In this paper, we present initial evidence that phylodynamic methods can be used to study cholera outbreaks at a regional level, and that they produce parameter estimates that are consistent with established methods. Our approach provides a common methodology for an early analysis of the model viability in the context of joint inference from different data sources. Given the complementary view offered by independent data sources, we anticipate that the analysis presented in this paper will find a widespread use in building joint hybrid epidemiological and genetic models which could help verify the main modelling assumptions.

## 2. Results

### (a) Integrated data from case counts, genomics and transportation data

**Figure 1.**
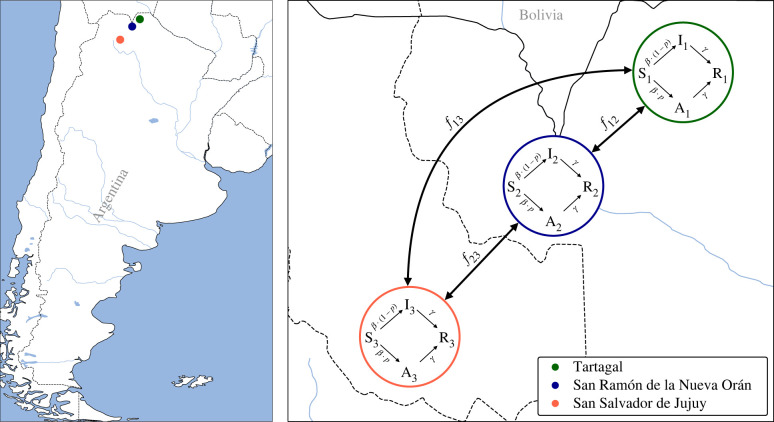
Meta-population model of the cholera transmission dynamics interlayed with a map of the northern Argentina. Left: three cities considered in our focused study are marked as follows: Tartagal—green circle, San Ramón de la Nueva Orán (in what follows, referred to as Oran)—blue circle—and San Salvador de Jujuy (in what follows, referred to as Jujuy)—orange circle. Right: the dynamics inside each city population is modelled using the Susceptible-Infected-Asymptomatic-Recovered (SIAR) model with seasonality modulated infection rate *β*(*t*), recovery rate *γ* and the parameter *p* representing the fraction of asymptomatic cases under the infection process. The amplitude of the infection rate is *β*_*s*_ for cities with a smaller population (Tartagal and Oran), and *β*_*l*_ for a larger city (Jujuy). Black arrows represent the migration flows of susceptible and asymptomatic individuals between cities, proportional to the flow rates *f*_*ij*_ for migration between locations *i* and *j*. A more detailed description of the model is provided in the Material and methods, as well as in the electronic supplementary materials, section C.

Cholera was first reported in Argentina in 1992, and subsequent cholera cases were reported until 1998 [26–30]. Out of the total 4281 cases reported, over 3500 *Vibrio cholerae* isolates were stored at INEI-ANLIS ‘Dr. Carlos G. Malbrn’, the national reference laboratory for Argentina, and a representative sub-sample of 532 of these isolates were previously whole-genome sequenced [16], see electronic supplementary materials, section A for more details. We sought to determine if there was agreement between epidemiological and genomic data. First, we pre-processed the dataset by keeping only the samples with O1 serogroup, specified date and a human origin. Second, we removed cities with insufficient genomic samples (less than 40 sequences). This left us with three target cities: Tartagal, San Ramón de la Nueva Orán (both in Salta province) and San Salvador de Jujuy (in Jujuy province) located within the northwest of Argentina (figure 1). Initial reports in 1992 indicated that cholera was first introduced into Argentina via this region from Bolivia, leading to a large outbreak from 1992 to 1993 [31].

In addition to the epidemiological and sequence data, we used publicly available data on domestic travel to estimate the movement of population between these three cities during the study period. Focusing on the two primary means of transportation, flights and buses, we were able to estimate the typical number of people travelling daily between the selected cities (for details see Material and methods and electronic supplementary materials, section B).

### (b) Modelling assumptions

We modelled the cholera transmission dynamics using a system of ordinary differential equations (ODEs) where the population is split into compartments representing individuals in different states of infection. Typical cholera models are a system of ODEs representing a modification of the classical Susceptible-Infected-Recovered (SIR)-type model [32] with varying degrees of complexity [17,22,33]. Here, we present a new, simple ODE cholera model that significantly advances estimation of key epidemiological parameters in two ways: (i) we focus on a minimalist representation which allows us to reliably infer model parameters from a limited amount of data while introducing the least amount of assumptions and (ii) we use a meta-population structure to leverage the spatial knowledge on reported cases and travel patterns that can represent the major spreading mechanism. We also do not consider re-infection in our models of localized outbreaks, as protective immunity against cholera has been estimated to last at least 3 years [2]. Prior to formally introducing our dynamic model, we discuss the main modelling assumptions behind our approach.

Many cholera models in the literature include an environmental compartment [17–21]. Such an environmental compartment is typically introduced to explicitly model the transmission of infection through a water source, and additionally describes the evolution of bacteria in a water source with temperature-dependent dynamics. From the fitting perspective, an environmental component may have a benefit to help the multi-year epidemic outbreak (see the span of observed cases in the electronic supplementary materials, figure S1) survive the period of cool temperatures when number of cases drop significantly, and re-occur when the temperature rises. During our initial model exploration, we tested an extension of our model that included an environmental compartment, finding that its inclusion did not significantly improve the quality of the fit, while at the same time it introduced additional parameters that needed to be inferred from data. For this reason and in order to keep the number of model parameters small, we do not explicitly include the environmental compartment in our model. Instead, the seasonal component of cholera transmission, well documented in [34–36], is included in a direct transmission parameter of our model. This direct contact parameter is an *effective* parameter describing the spread of cholera which includes all potential transmission channels, similarly to an approach used in [22]. Previously, seasonally modulated transmission parameters was suggested in a fully theoretical framework in [37], but it was not applied to empirical data.

Our second key assumption is related to the presence of an asymptomatic population, which does not display any strong symptoms, but nevertheless contributes to the infection spread via migration. This population is not directly observed, but contributes to the cholera dynamics via a dedicated compartment *A*. The associated parameter *p* describes the fraction of infected population which falls into the *A* compartment upon infection, while the rest of the population falls into the *I* compartment which describes the symptomatic population. In spite of the general agreement that a significant number of individuals infected by cholera display no apparent symptoms, the literature is not conclusive about the proportion of asymptomatic carriers. For instance, the World Health Organization (WHO) [23] suggests that between 1% and 25% of infected cases are asymptomatic, [24] estimates *p* closer to 50% and according to [25], the asymptomatic population represents the majority of cases. Therefore, we treat *p* as one of the key free parameters in our model which will need to be inferred from data. We further assume that the transmission dynamics between cities through migration is mediated by the asymptomatic carriers only. This assumption is incorporated into the meta-population model through a migration term which is proportional to the number of asymptomatic individuals in the population, and is appropriately normalized so that the city populations do not change. Additionally, these migration terms are informed by independently estimated travel rates, as we discuss below. These migration terms link epidemic trajectories in different cities and thus facilitate the identification of the parameter *p* related to the fraction of asymptomatic cases upon infection.

### (c) Meta-population model

Here, we formally summarize our dynamic meta-population model. The cholera dynamics in each of the three cities is described using a homogeneous SIR-like SIAR model, linked by a flow of asymptomatic infected individuals between cities. More precisely, we divide the population of each city into four compartments: susceptible (S), infected symptomatic (I), infected asymptomatic (A) and recovered (R). The migration mechanism allows for a mixing of different city populations with two conditions: (i) symptomatic infected individuals do not move between locations, (ii) travelling on average does not change the population of each city. A schematic of the structure of the meta-population model and the details of the single-city model are shown in figure 1. The exact system of ordinary differential equations used to build the model is described in detail in the electronic supplementary materials, section C.

The model contains a total of 10 epidemiological, travel and demographic parameters. The epidemiological parameters are the recovery rate *γ*, which represents the inverse of expected days to recovery, the parameter *p*, which represents the fraction of asymptomatic cases emerging upon infection, and the infection rate *β*(*t*) modulated by a time-dependent function reflecting the seasonal changes. Importantly, while the seasonality itself is assumed to be the same for all three geographically close cities, the amplitudes *β*_*s*_ and *β*_*l*_, respectively representing the smaller population cities Tartagal and San Ramón de la Nueva Orán, and the larger population San Salvador de Jujuy, may be different. The transmission amplitudes *β*_*s*_ and *β*_*l*_ can *a priori* take different values, for instance due to an expectation of a different quality healthcare and infrastructure in both types of cities. An example of a better infrastructure includes access to cleaner water, while higher-quality healthcare is exemplified by a quicker response, isolation of patients and treatment, both potentially resulting in lower infection rates. Additionally, the model parameters include the initial demographic structure of all four compartments in each location. Finally, the model contains a set of migration parameters *f*_*ij*_ describing the flow of people between the cities. The procedure for estimating the migration parameters is given in the electronic supplementary materials, section B. A more detailed description of the fixed and free parameters is included in the Material and methods, as well as in the electronic supplementary materials, section C.

### (d) Estimation of model parameters from case-count data

Using a least-squares-based estimator minimizing the error between the case-count data and model predictions (see Material and methods for more details), we estimated the transmission rates, seasonality parameters, initial conditions and asymptomatic fraction *p*. One of the main challenges faced by the fitting of our continuous meta-population SIAR model was the fact that the case counts are not reported continuously in the dataset, but instead appear as discrete peaks at certain sampling days. This can be related to several factors, including reporting aggregated data only on certain days, a surveillance procedure only taking samples on certain days or gathering data from different locations. To address this challenge, we proposed a sampling model which establishes a correspondence between the continuous model and the case counts sampled at specific dates by looking at a cumulative number of cases between subsequent sampling dates (see Material and methods and electronic supplementary materials, section D).

The results of the inference procedure are presented in table 1 for the key model parameters *p*, *β*_*s*_ and *β*_*l*_ (see electronic supplementary materials, section E). In the absence of ground truth, we use the following approach to estimate the uncertainty of our inference procedure. We construct a synthetic model with planted ground-truth parameters equal to the parameters inferred from data. Then, by generating counting noise on the same sampling dates as the ones that appear in the real data, we can construct synthetic datasets which have the same properties as the original case count data, but with the advantage that these datasets now come with a planted ground truth. We run our estimator on many instances of synthetic data with different noise realizations and compare the inference results with the planted parameters of the synthetic model. This procedure allows us to reliably estimate the uncertainty bounds of our inference procedure. Previously, a similar procedure for estimating the uncertainty in the absence of ground truth has been used in other applications involving statistical inference [38,39]. To check robustness with respect to the (unknown) counting noise, we consider the estimation error for noise generated from several families of probability distributions, and report the standard deviation results averaged over these families. This approach bears similarity to the *parametric bootstrap* [40], or simple re-sampling of residuals. The details of this procedure are given in the electronic supplementary materials, section E.

**Table 1.** Model parameters inferred from the case-count data, together with their single standard deviation uncertainty averaged over several families of statistical counting noise (top panel), and from the genetic sequence data with 95% highest posterior density intervals (HPDI) (bottom panels under two different inference settings, with *p* fixed or free). Owing to scarcity of genetic data, only parameters shown are inferred from data, whereas the others (*ϕ*, *m*_0_, *A*(0)) are fixed to values inferred from case-count data, and shown in electronic supplementary materials, table S3.

data (setting)	parameter	lower	inferred	upper
case counts (*ϕ*, *m*_0_, *A*(0), *β*_*s*_, *β*_*l*_, *p* free)	*β*_*s*_	0.144	0.157	0.170
	*β*_*l*_	0.148	0.155	0.162
	*p*	0.04	0.32	0.60
genetic sequence (*β*_*s*_, *β*_*l*_ free, others fixed)	*β*_*s*_	0.160	0.161	0.162
	*β*_*l*_	0.162	0.163	0.164
genetic sequence (*β*_*s*_, *β*_*l*_, *p* free, others fixed)	*β*_*s*_	0.160	0.161	0.162
	*β*_*l*_	0.162	0.163	0.164
	*p*	0.85	0.94	0.99

**Figure 2.**
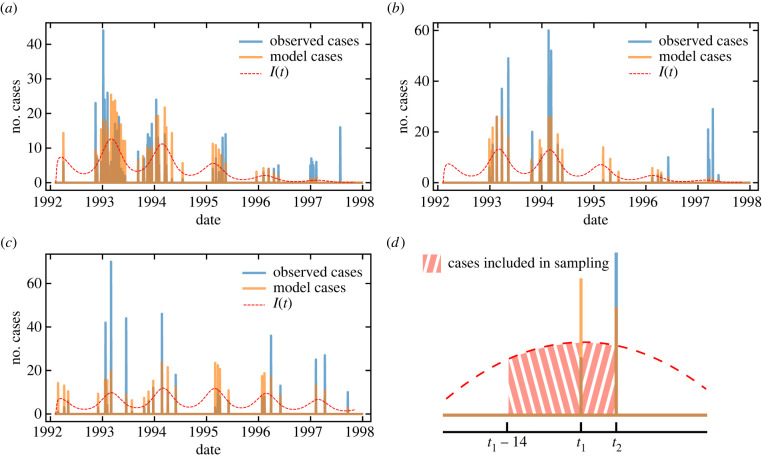
Comparison of the case-count data (blue bars) and the samples obtained from the model with the inferred parameters (orange bars) for three cities: (*a*) Tartagal, (*b*) Oran and (*c*) Jujuy. The red dashed line represents the number of active infected symptomatic cases according to the predictions of our continuous meta-population SIAR model. In the real dataset, the case counts are not reported every day, but instead appear as discrete peaks at certain sampling days, which complicates the fitting of the continuous model. For this reason, we propose a correspondence between the continuous model and the case counts sampled at specific dates. The panel (*d*) explains the sampling model we use in our fitting procedure. We assume that the case counts reported on a given date correspond to a cumulative number of cases predicted by the continuous model between the minimum of the current and the previous sampling dates and 14 days before the current sampling date. This cut-off cumulative horizon represents double the expected recovery period (fixed to 7 days, as explained in the Material and methods). The differences between the heights of orange bars and blue bars represent residuals between the predictions of our model and the data. A more detailed explanation of the sampling procedure and the study of the impact of the choice of the sampling horizon is presented in the electronic supplementary materials, sections D and I, respectively. (*a*) Tartagal, (*b*) San Ramón de la Nueva Orán (Oran), (*c*) San Salvador de Jujuy (Jujuy) and (*d*) sampling procedure example.

A comparison of the true case count data and the samples obtained from the model with the inferred parameters is shown in figure 2. Despite some discrepancies observed for the highest case-count peaks, our simple model is able to adequately describe the variations present in the data, including the seasonal character of the cholera outbreak in Argentina. The obtained seasonality of the transmission rate highly correlates with seasonal temperature variations in the analysed cities (see electronic supplementary materials, section F), even though such information was not explicitly implemented in the model and was not provided to the inference algorithm. The model suggests that cholera from this initial outbreak dies out after 1997, whereas the cases are not only reported in the dataset after 1997, but they even see an increase in comparison to the previous years. This is consistent with a hypothesis that the nature of the further peaks may have been significantly influenced by external factors such as the Mitch hurricane in 1998 or El Niño in 1997–1998 [41] (see Material and methods).

Our results are highly robust. We successfully tested our fitting procedure with synthetic data generated with different types of noise (see electronic supplementary materials, section E). We have also studied the scenario of a non-centred counting noise distribution corresponding to a significant under-reporting of the case counts. In the electronic supplementary materials, section G, we show that this scenario is unrealistic as it leads to an unreasonably large number of predicted infected individuals, of the order of the whole city population. Moreover, we tested the sensitivity of our procedure with respect to the mis-specification of the inferred migration rates (see electronic supplementary materials, section H) and different sampling horizons (see electronic supplementary materials, section I). Finally, we investigated how much the length of the case-count time series affected the estimated parameters (see electronic supplementary materials, section J). We conclude that despite many sources of potential discrepancies, estimates are robust to reasonable levels of model mis-specification.

### (e) Estimation of model parameters from genetic sequence data

**Figure 3.**
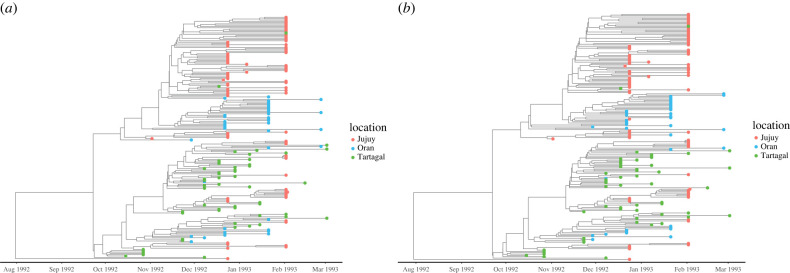
The time-scaled phylogenetic tree of sequenced cholera cases sampled from Jujuy, Oran and Tartagal. Tip colours indicate the city of sampling and position along the *x*-axis indicates the date of sampling. The phylodynamic analysis is restricted to a single outbreak period from 13 November 1992 to 2 March 1993 that contained the majority of the sequence data (see Material and methods). This maximum-likelihood tree topology suggests a rapidly growing epidemic with multiple transmissions between the three locations, which suggests the key role played by the migration. A migration mechanism is included in our meta-population model through travel of asymptomatic individuals that link the dynamics in different cities. (*a*) *p* fixed and (*b*) *p* free.

We used phylodynamic methods specifically developed to fit epidemiological transmission models to sequence data [42] to estimate the transmission rates and the asymptomatic fraction parameter *p* from the genetic sequence data, using the same meta-population SIAR model (see Material and methods and electronic supplementary materials, section K, for a description of the data and the details on the inference procedure). The maximum clade-credibility trees from models with and without *p* fixed are shown in figure 3. The tree typologies suggest an intermixed outbreak with evidence of multiple transmissions occurring between the three cities, which provides an additional justification for the inclusion of the migration mechanism in our model. Likewise, the long branch lengths are indicative of a rapidly growing epidemic which is consistent with results from both the case-count model and the inferred transmission parameters based on the genetic sequence data, as we show next.

The principal goal of this paper consists of verifying the level of congruity of model parameters inferred from case-count and genetic data. In order to check for consistency between the two datasets, we consider two different settings for inference from the genetic data. Under setting 1, we fix *p* to the expected value inferred from the case-count data, and estimate the transmission rates *β*_*s*_ and *β*_*l*_. Under setting 2, we leave all three parameters *β*_*s*_, *β*_*l*_ and *p* free, and infer them from the genetic data. In both settings, all other parameters were either fixed to the MLE values from the case-count data or constrained by a prior centred at the maximum likelihood estimator (MLE) values. The results of the inference procedure under both settings is given in table 1, along with the uncertainty bounds with 95% highest posterior density interval (HPDI).

**Figure 4.**
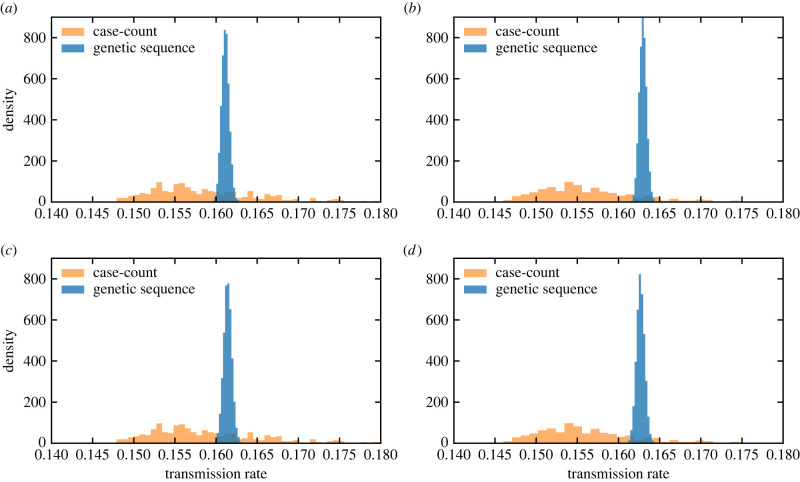
Comparison of the posterior densities for *β*_*s*_ and *β*_*l*_ under two settings of inference from genomic data with the densities sampled from the model with parameters inferred from the case-count data. Under setting 1, we estimate *β*_*s*_ and *β*_*l*_ (the fraction of asymptomatic infections *p* is fixed to the value inferred from the case-count data), and under setting 2, we estimate *β*_*s*_, *β*_*l*_, as well as the free parameter *p*. All other parameter values were set to the maximum-likelihood estimates from the case-count model. The orange transparent bars in the background represent the synthetic densities obtained with model parameters inferred from the case-count data. Compared with the estimates of transmission rates from the case-count model, the point estimates of *β*_*s*_ and *β*_*l*_ from the genetic sequence data are generally higher but still consistent within the bounds of uncertainty. (*a*) *β*_*s*_ (setting 1, *p* fixed), (*b*) *β*_*l*_ (setting 1, *p* fixed), (*c*) *β*_*s*_ (setting 2, *p* free) and (*d*) *β*_*l*_ (setting 2, *p* free).

A comparison of the posterior densities for *β*_*s*_ and *β*_*l*_ under both phylodynamic inference settings with the density sampled from the model with parameters inferred from the case-count data are shown in figure 4. Compared with the estimates of *β*_*s*_ and *β*_*l*_ from the case-count model (table 1), the point estimates from the phylodynamic model are generally higher but still consistent within the bounds of uncertainty. We note that the uncertainty bounds from inference on the genetic data are tighter compared with the confidence intervals obtained from inference on the case count data. Significant caution should be taken in interpreting the uncertainty of the point estimates for the genetic data as the narrowness of the HPDI is likely due to the need to fix most parameters for computational concerns. All parameters besides *β*_*s*_, *β*_*l*_ and *p* are either fixed or constrained by a narrow prior density, and the fixed migration terms impose strong constraints on the parameter *p*. The phylodynamic model also infers a higher mean transmission rate *β*_*s*_ for the smaller cities of Tartagal and Oran than *β*_*l*_ for the larger city of Jujuy. Although the estimate of *p* (0.94, 95% HPDI 0.85, 0.99) under setting 2 was quite different from the case-count model, overall the *β*_*s*_ and *β*_*l*_ parameters were robust to the fixed value of *p* = 0.32 (setting 1) and the estimated value of *p* = 0.94 (setting 2). Posterior trajectories for settings 1 and 2 are discussed in the electronic supplementary materials, section L.

## 3. Discussion

Our results demonstrate that phylodynamic methods applied to single-source, local cholera outbreaks produce different parameter estimates, but show a significant level of consistency with traditional epidemiological models based on case-count data, especially taking into account results from a number of sensitivity experiments that we conducted. Our study found that point estimates of the transmission rates based on case-count and migration data alone produces consistently lower estimates of the transmission rates compared with the genetic sequence data. While the estimates were within the uncertainty bounds for estimated transmission rates from the case-count data, the genetic sequence data found larger estimated outbreak sizes. In part, this discrepancy can be due to the fact that the genetic sequence data only came from a single seasonal cycle and was therefore not constrained to maintain a sufficient susceptible population to produce outbreaks in subsequent years: we have observed a similar impact of the data length on the case-count inference in electronic supplementary materials, section J. The discrepancy could also represent a real difference of opinion in the data streams. Models based on case counts can only ‘see’ the dynamics that are present in the observed cases, where biased or incomplete sampling can lead to underestimation of the extent of an outbreak. However, the phylogenetic structure of pathogen genetic sequence data is fundamentally shaped by the transmission dynamics that give rise to the data regardless of how many cases were actually discovered, that is, phylodynamic methods can reveal a more complete picture of transmission dynamics given incomplete data.

The main difference in the image of the outbreak provided by these different data are the difference in uncertainty in the transmission rate estimates and the proportion of asymptomatic infections. The high level of certainty in the genetic estimates of *β*_*s*_ and *β*_*l*_ should be regarded more as an artefact of necessary trade-offs made for computational efficiency, as well as of constraints on key parameters due to a smaller sample size and fixing of other model parameters. At present, phylodynamic methods are only really feasible for relatively small datasets with relatively simple models. We found that using time-variable parameters also significantly increased the computational cost of a joint sampling of the space of phylogenetic trees and model parameters. Future development of phylodynamic methods for bacterial pathogens with complex transmission dynamics should focus on resolving these technical areas for more robust phylodynamic inference. The genetic sequence data also found a much higher proportion of asymptomatic infections *p* than the case-count data. This is partially due to limits in sampling frame for the genetic sequence data. Because we could only fit the model to one year of sequence data, there was little cost to infecting a very large fraction of the population in a single year, which is what happened when the fraction of asymptomatic infections *p* was not constrained (see setting 2 in table 1). We observed a similar increase in the estimate of the fraction of asymptomatic infections *p* in the case-count inference when restricting the case-count data to a 1 year span (see electronic supplementary materials, section J). The model as applied to the genetic sequence data also assumed that the migration rates were fixed to the values inferred directly from the data. While we found this to be a necessary assumption in our computational pipeline, it is possible that the genetic sequence data favours an overall faster migration process than is supported by the travel data and compensates by simply assuming a higher asymptomatic fraction: we observed a similar effect in our analysis of sensitivity to a mis-specification of migration flows *f*_*ij*_ reported in electronic supplementary materials, section H. Regardless of a particular inferred or assumed value of *p*, we found that the transmission amplitudes *β*_*s*_ and *β*_*p*_ were very robust to different values of the fraction of asymptomatic infections (table 1).

In this paper, we demonstrated that, taken independently, case-count and genetic sequence data provide a complementary view of cholera transmission dynamics at the city/regional level. This analysis represents a necessary data consistency check prior to building a joint framework for inference from different data sources. The next challenge for integrating genetic sequence data into cholera modelling projects is joint inference on both case-count time series and genetic sequence data. Allowing both case-count and genetic sequence data to contribute to a model would allow us to better estimate parameters such as the level of under-reporting of cholera cases (referred to as statistical counting noise in electronic supplementary materials, sections E and G in our study), the fraction of asymptomatic infections *p*, and possible differences in asymptomatic versus symptomatic transmission at the population level. Developing a population of generalized cholera models that can integrate case-count, genetic sequence and migration data into a single picture of cholera outbreak dynamics will give cholera researchers more power to differentiate between different modelling assumptions, for example, more carefully elucidating the role of environmental versus direct transmission in sustaining cholera transmission in multi-year outbreaks.

## 4. Material and methods

### (a) Fixed model parameters

We infer the free parameters of the model from the data. However, some of the parameters are not independent, or are well documented, and hence can be fixed. The model parameters that are fixed during our inference procedure include the recovery rate *γ*, the city populations *N*_*i*_, the initial values of compartments *I*_*i*_(0), *R*_*i*_(0) and the migration parameters *f*_*ij*_. The recovery rate of cholera is well documented in the literature [23,43–45] and equal to $\frac{1}{7}$, which represents the average period of 7 days to recovery. We use available historical population data to set the total population size *N* of each city: *N* = 4.4 × 10^4^ in Tartagal, *N* = 5.1 × 10^4^ in San Ramón de la Nueva Orán and *N* = 2 × 10^5^ in San Salvador de Jujuy. The initial values of *I*_*i*_(0) and *R*_*i*_(0) for all cities *i* are taken as zero, since we follow the assumption that the infection was most likely introduced in Salta and Jujuy provinces by a single asymptomatic migration event [31]. As a consequence, for all locations *i* in the meta-population model, *S*_*i*_(0) = *N*_*i*_ − *A*_*i*_(0), where *A*_*i*_(0) is a free parameter which is inferred from the case-count data, but is assumed to be the same for all three cities, i.e. $A_{i}(0)=A(0)\;\forall i$. Further following the documented suggested sequence of events preceding the initial epidemic outburst in Salta and Jujuy provinces [31], we assume that 5 ≤ *A*(0) ≤ 15. Lastly, the migration rates *f*_*ij*_ for a pair of locations (*i*, *j*) are fixed based on available documented and extrapolated data about domestic travel in Argentina. Values of *f*_*ij*_ used in the model can be found in the electronic supplementary materials, section B.

In addition, a certain choice for the starting and ending dates is needed to connect the model predictions with the available case-count data. The first case in Salta province was sampled on 10 February 1992 in our dataset, whereas according to Mazzafero *et al*. [31], the migration of asymptomatic cases into Salta province started around the first week of February 1992. As a result, we use an intermediate date of 8 February 1992 as the starting date in our inference procedure. The last cases sampled in Argentina and present in our dataset are from 2002 (2000 in Salta province), however, as suggested in [41], major external events such as the Mitch hurricane in 1998 or El Niño in 1997–1998 significantly affected the dynamics of cholera in South America. In order to omit this potentially strong influence of external events on our data, we only use the available data up until 1998.

### (b) Inference of model parameters from case-count data

Epidemiological case data are subject to different sources of uncertainty and statistical noise: missing observations, delayed reporting, specific surveillance procedure, technical and laboratory errors, etc. While the observed number of cases can be higher or lower than the true number of infected individuals in a given period, it is reasonable to assume that a delay exists between the occurrence and observation of a case for both objective (e.g. lack of nearby facilities) and administrative (e.g. delay in reporting) reasons. As a consequence of such a delay, the case counts appearing in the data appear as discrete peaks at specific days. To account for this, we assume that in each city, raw data represent a sampling of its infected population, where the number of cases reported on a specific date consists of a cumulative number of symptomatic cases observed between the current and the previous sampling dates, unless the previous sampling date appears more than 14 days before the current data (in which case the sampling horizon is fixed to 14 days). This cut-off cumulative horizon represents the double of expected recovery period 1/*γ* (which is fixed to 7 days, as explained above). A graphical representation of this procedure is shown in figure 2*d*. Details of the assumed sampling procedure are described in electronic supplementary materials, section D and additional experiments showing the impact of choosing different sampling horizons is presented in electronic supplementary materials, section I. This procedure allows us to directly compare the reported case data with the predictions of our continuous model using the sampling procedure described above.

In most of the presented results, we infer the model parameters by minimizing the average square error between the modelled and observed case counts. In a maximum-likelihood setting, this is equivalent to the assumption of normally distributed statistical noise on the case counts. In our analysis with synthetic data, we found this assumption to be well justified (electronic supplementary materials, section E). In our inference procedure, the average square error is minimized using the Limited-memory Broyden–Fletcher–Goldfarb–Shanno algorithm with bounded constraints (L-BFGS-B) [46]. Given that the resulting optimization problem is highly non-convex, we used the warm-starting strategy by initializing the algorithm multiple times, with different initial conditions, in order to increase the probability of finding the global minimum. The code used to obtain all our results is available at [47].

### (c) Inference of model parameters from pathogen sequence data

Publicly available alignment files from a previously published study on whole-genome sequencing of *Vibrio cholerae* isolates from Argentina [16] were downloaded from FigShare [48]. Here, we used the alignment data using the Peruvian strain A1552 isolated in 1991 as the reference genome [16]. We used the PhyDyn library [49] for the BEAST2 phylogentics platform [50] to fit the cholera ODE model to the genetic sequence data, see electronic supplementary materials, section K for implementation details. We restrict the phylodynamic analysis to a single outbreak period from 13 November 1992 to 2 March 1993 that contained the majority of the sequence data, resulting in 55, 41 and 90 sequences for Tartagal, San Ramón de la Nueva Orán and San Salvador de Jujuy, respectively. Although we set the starting time for the outbreak *t*_0_ to 8 February 1992 to be consistent with the case-count model, we exclude the 11 sequences sampled between March and April of 1992 (see electronic supplementary materials, section A) to improve computational efficiency. We use the known sampling times of each sequence with the Hasegawa, Kishino, Yan (HKY) substitution model [51] with Gamma-distributed, site-specific substitution rates assuming a strict clock to time scale the tree. The initial starting tree was fixed to its maximum-likelihood topology inferred using IQtree2 [52]. We also found that, rather than fixing the migration rates to their MLE values, the sampling of the joint tree and model space was slightly more efficient if we used a Normal prior with mean at the MLE value from the case-count data and standard deviation equal to the square root of the mean.

In order to check a consistency between the phylogenetic and traditional epidemiological data, we use two different settings for inference from the genomic data. Under setting 1, we fix *p* to the expected value inferred from the case count data, and estimate the transmission rates *β*_*s*_ and *β*_*l*_. Under setting 2, we infer all three parameters *β*_*s*_, *β*_*l*_ and *p* from the genomic data. Under both settings, inference procedures were run in such a way that all effective sample sizes were greater than 500 with the first 10% of samples being removed as burn-in. The details on phylodynamic inference under both settings are provided in the electronic supplementary materials, section L.

## Data Availability

The case-count data used in this study is available at [47]. Publicly available alignment files from a previously published study on whole-genome sequencing of *Vibrio cholerae* isolates from Argentina [16] were downloaded from FigShare [48]. In this study, we used the alignment data using the Peruvian strain A1552 isolated in 1991 as the reference genome. All other data that support the plots within this paper and other findings of this study are available from the authors on reasonable request. The data are provided in electronic supplementary material [48].
